# Social Cognition in Schizophrenia and Autism Spectrum Disorders: A Systematic Review and Meta-Analysis of Direct Comparisons

**DOI:** 10.3389/fpsyt.2018.00504

**Published:** 2018-10-24

**Authors:** João Miguel Fernandes, Rute Cajão, Ricardo Lopes, Rita Jerónimo, J. Bernardo Barahona-Corrêa

**Affiliations:** ^1^Department of Psychiatry and Mental Health, NOVA Medical School|Faculdade de Ciências Médicas, Centro Hospitalar de Lisboa Ocidental, Lisbon, Portugal; ^2^Department of Psychiatry and Mental Health, Centro Hospitalar Tondela-Viseu, Viseu, Portugal; ^3^Instituto Universitário de Lisboa (ISCTE-IUL), CIS-IUL, Lisbon, Portugal; ^4^CADIN—Neurodevelopment, Cascais, Portugal; ^5^Champalimaud Clinical Centre, Champalimaud Centre for the Unkown, Lisbon, Portugal; ^6^Champalimaud Research, Champalimaud Centre for the Unknown, Lisbon, Portugal

**Keywords:** autism spectrum disorders, Asperger syndrome, schizophrenia, social cognition, theory of mind, emotion perception

## Abstract

**Background:** Deficits in social cognition are well-recognized in both schizophrenia and autism spectrum disorders (ASD). However, it is less clear how social cognition deficits differ between both disorders and what distinct mechanisms may underlie such differences. We aimed at reviewing available evidence from studies directly comparing social cognitive performance between individuals with schizophrenia and ASD.

**Methods:** We performed a systematic review of literature up to May 22, 2018 on Pubmed, Web of Science, and Scopus. Search terms included combinations of the keywords “social cognition,” “theory of mind,” “autism,” “Asperger,” “psychosis,” and “schizophrenia.” Two researchers independently selected and extracted data according to PRISMA guidelines. Random-effects meta-analyses were conducted for performance on social cognitive tasks evaluating: (1) emotion perception; (2) theory of mind (ToM); (3) emotional intelligence (managing emotions score of the Mayer-Salovey-Caruso Emotional Intelligence Test); and (4) social skills.

**Results:** We identified 19 eligible studies for meta-analysis including a total of 1,040 patients (558 with schizophrenia and 482 with ASD). Eight studies provided data on facial emotion perception that evidenced a better performance by participants with schizophrenia compared to those with ASD (Hedges' g = 0.43; *p* = 0.031). No significant differences were found between groups in the Reading the Mind in the Eyes Test (8 studies; Hedges' g = 0.22; *p* = 0.351), other ToM tasks (9 studies; Hedges' g = −0.03; *p* = 0.903), emotional intelligence (3 studies; Hedges' g = −0.17; *p* = 0.490), and social skills (3 studies; Hedges' g = 0.86; *p* = 0.056). Participants' age was a significant moderator of effect size in emotion perception and RMET analyzes, with larger differences favoring patients with schizophrenia being observed in studies with younger participants.

**Conclusions:** The instruments that are currently available to evaluate social cognition poorly differentiate between individuals with schizophrenia and ASD. Combining behavioral tasks with neurophysiologic assessments may better characterize the differences in social cognition between both disorders.

## Introduction

### Rationale

Social cognition concerns the detection, processing and use of social information to regulate interpersonal functioning and effective social behavior (1, 2). Schizophrenia and autism spectrum disorders (ASD) are two conditions characterized by significant impairments in social cognition (1, 3). Impaired social cognition is a major driver of poor psychosocial functioning in both disorders and has been increasingly considered as one of the key treatment targets in psychosocial and biological therapeutic interventions (4–6).

In schizophrenia, social cognition impairments have mostly been described in the following domains: (1) emotion perception, defined as the ability to identify emotions, for example from a facial expression or tone of voice; (2) theory of mind (ToM), defined as the ability to infer other people's mental states (their intentions, desires or beliefs); (3) attributional style, defined as the way by which individuals explain the causes of positive and negative events (i.e., by attributing responsibility either to themselves, to others or to the situation); and (4) judgment, including the ability to extract meaning from environmental information, and the processing bias known as “jumping to conclusions,” which refers to the tendency to formulate definitive judgements based on insufficient confirmatory evidence (4, 7). In turn, social cognition deficits in ASD have been primarily defined based on a broader concept of ToM as the ability to reflect on one's own and others' mental states (mentalizing) (8). Therefore, the definition of ToM that is most prevalent in ASD literature encompasses not only the ability to take the perspective of others and to interpret others' beliefs, desires or intentions (frequently defined as “cognitive ToM”), but also emotions (“emotional or affective ToM”) (8, 9). ToM has been further classified, within the context of both ASD and schizophrenia, into first-order ToM (the ability to infer what another person is thinking about an objective situation) and second-order ToM (the ability to infer what another person is thinking about what a third person is thinking about an objective situation) (5, 10).

Patients with schizophrenia and ASD have consistently been shown to perform worse than neurotypical controls in social cognitive tasks (11–13). In a meta-analysis of 37 studies evaluating mentalizing capacity in adult patients with schizophrenia or ASD in comparison to neurotypical controls, both groups showed similar levels of significant impairment in verbal mentalizing capacity (intention/belief inference) and visual mentalizing capacity (assessed by the Reading the Mind in the Eyes Test [RMET]). The schizophrenia group showed a trend toward greater impairment of verbal mentalizing ability than of visual mentalizing ability, while participants with ASD showed similar levels of impairment in both tasks (11). In ASD, male gender was associated with greater impairment of cognitive ToM ability at a trend level, and mentalizing ability was found to be independent of age (11). In another meta-analysis, studies using a Triangles Animation Task designed to assess attribution of mental states were reviewed in an effort to identify differential social cognition deficits between schizophrenia and ASD (12). However, this analysis only included one direct comparison between patients with schizophrenia and ASD, with the remaining 20 studies comparing the clinical groups with neurotypical controls. In their respective comparisons with neurotypical controls, the ASD group had generally larger standardized mean differences than the schizophrenia group in terms of ability to appropriately describe the animations, with similarly sized standardized mean differences with respect to deficits in intentionality detection. Moreover, patients with first-episode psychosis performed better than patients with longer lasting schizophrenia, suggesting that duration of schizophrenia may be associated with a reduction in mentalizing abilities (12). More recently, a meta-analysis (published as an abstract) of 74 studies in schizophrenia (3,555 cases) and 22 studies in ASD (810 cases), also confirmed the existence of significant ToM deficits in both clinical groups (13). Inference of intentions from verbal tasks was a significant area of deficit for patients with schizophrenia, but not for the ASD group. The latter, in turn, showed markedly impaired ability to understand the meaning of indirect speech. Additionally, positive symptoms were found to modulate the magnitude of ToM deficits in schizophrenia (13).

With respect to the “jumping to conclusions” dimension of social cognition, although it has typically been studied as a specific deficit of schizophrenia, at least one study by Brosnan et al. found that ASD subjects show a more circumspect reasoning bias (that is, a need to gather more data before a decision is made), which is the opposite pattern of the jumping to conclusions reasoning bias observed in schizophrenia (14). The study authors concluded that these findings are consistent with the Autism-Psychosis Model proposed by Crespi and Badcock (15), which proposes that patients with autism and schizophrenia show opposite patterns of response in social cognitive tasks, with underdeveloped social cognition in ASD and hyper-developed social cognition in psychotic disorders. A similar formulation has been proposed by Simon Baron-Cohen in his Empathizing-Systemising Theory (16), according to which ASD subjects show high Systemising and deficits in Empathizing, while the opposite pattern (low Systemizing and high Empathizing scores) is associated with higher levels of psychotic experiences and jumping to conclusions bias (15).

All in all, it remains unsettled whether or not schizophrenia and ASD differ in terms of social cognitive performance (1), with their shared genetic risk, partly overlapping pathogenic mechanisms (17) and phenomenological proximity (particularly insofar as social interaction deficits, communication difficulties and restricted interests are concerned) (18), fuelling an ongoing debate on whether the two conditions lie on the same neurodevelopmental and phenotypic continuum (17–19). The available literature has reached contradictory conclusions on this issue, with the few existing meta-analyses allowing for indirect comparisons at best. This may be inadequate to compare social cognitive performance in these two populations because of methodological differences across studies (20), in addition to other sources of inconsistency such as the inclusion of studies with small sample sizes, and the use of different tasks or different task versions, sometimes using different instructions, cueing and rating systems (12). Another unsettled issue regards the possibility that the instruments currently available to assess social cognition, especially emotion perception and ToM, may have poor discriminatory power between schizophrenia and ASD.

### Objectives

We set out to review studies that performed head-to-head comparisons of social cognition in subjects with ASD and with schizophrenia. Our main goal was to identify differences in social cognitive performance between patient groups that could help characterize the specific social cognition impairments of each disorder. Understanding how social cognition differs between schizophrenia and ASD, and what underlying mechanisms explain such differences, may help develop disorder-tailored interventions which may potentially improve outcomes, as targeted social cognitive interventions have been shown to be especially effective in improving emotion perception and ToM (21).

### Research question

The research questions for this review were: (1) do direct comparisons of patients with schizophrenia and ASD show any differences in social cognitive performance? (2) do these differences in social cognition ability between patients with schizophrenia and individuals with ASD contribute to our understanding of the specific deficits and mechanisms that underlie social cognitive impairments in both disorders?

## Materials and methods

### Study design

We conducted a systematic literature review to identify studies comparing social cognition between patients with schizophrenia and patients with ASD. Comparative meta-analyses were performed for those social cognition dimensions that were directly compared between patients with schizophrenia and patients with ASD in at least 3 individual studies.

### Participants, interventions, comparators

We reviewed studies including groups of patients with schizophrenia spectrum disorders (schizophrenia, schizoaffective disorder, schizophreniform disorder, schizotypal personality disorder, first-episode psychosis, delusional disorder, and psychosis not otherwise specified) and groups of patients with ASD (autism, Asperger's syndrome, and pervasive development disorders), regardless of age or gender. We included any study comparing social cognition across these two groups of patients.

### Systematic review protocol

The identification and selection of studies was conducted according to PRISMA guidelines. The following inclusion criteria were considered for the selection of studies for the meta-analyses:

- Original articles in English, French, German, Portuguese or Spanish, regardless of publication date or country of origin;
- Studies including human populations;
- Any clinical studies directly comparing social cognitive performance between groups of patients with schizophrenia spectrum disorders and groups of patients with ASD.

### Search strategy

The search was performed on Web of Science, Scopus and Pubmed and the search strings used were formed from combinations of the keywords “social cognition,” “theory of mind,” “autism,” “Asperger,” “psychosis,” “schizophrenia” and the Boolean operator AND. The search was concluded on May 22, 2018.

After eliminating duplicates using Mendeley library tools, two researchers reviewed the list of articles separately, selecting eligible reports according to the criteria defined. Abstracts from scientific meetings and conference proceedings were not considered eligible for meta-analysis, due to the frequently incomplete reporting of quantitative data and the risk of double inclusion of individual subjects in cases where conference proceedings were followed by regular publication of full articles in scientific journals at a later time.

### Data extraction

Two researchers extracted the following data from each eligible study: author and publication year, number of participants in the schizophrenia and ASD groups, mean age of each group, gender distribution of each group, mean intelligence quotient (IQ) of each group, psychometric outcome measures, summary of psychophysiological comparisons (where available), and any relevant additional information.

Psychometric outcome measures were classified according to the following social cognition dimensions: (1) emotion perception; (2) ToM (inferencing); (3) emotional intelligence; and (4) social skills. The outcome measures (tasks) that were used to assess each social cognition dimension are listed on Table 1. For each outcome measure from each eligible study we extracted raw group data (mean and standard deviation). When these were not provided, we extracted data from tests of differences (*t*-value, or *F*-value from Analysis of Variance tests). Data were extracted either directly from the text and tables or extrapolated from figures. In the latter situation values (mean and standard deviation) were extracted using Adobe Acrobat Reader measurement tools. To account for measurement error, each value from each figure was measured five times, and the mean value computed. In cases where data included in the original manuscript were insufficient, we contacted the corresponding author to request further information.

**Table 1:** Social cognition dimensions and outcome measures evaluated in the meta-analyses.

**Social cognition dimension**	**Outcome measure (studies using each outcome measure indicated within brackets)**
Emotion perception	Penn Emotion Recognition Task (ER-40) (3, 22) Social Scenes Task (Face Present Condition Score) (23) Emotions in Context Task (Faces in Isolation Score) (24) Developmental Neuropsychological Assessment NEPSY-II (Affect Recognition Subscale Score) (25) Facial Affect Recognition based on Ekman & Friesen (26, 27)
	Frankfurt Test for the Recognition of Facial Affects - Face Test (28)
Theory of Mind (inferencing)	Reading the Mind in the Eyes Test (RMET) (9, 27–33) Modified Advanced Theory of Mind Test (9) Movie for the Assessment of Social Cognition (MASC) (19) Hinting Task (30) Triangles Animation Task (27, 32) Social Reciprocity Scale (Cognition Subscale) (34) Yoni Task (Cognitive Subscale) (35) Comic Strips Task (36) Developmental Neuropsychological Assessment NEPSY-II (Verbal ToM Subscale) (25)
Emotional Intelligence	Mayer–Salovey–Caruso Emotional Intelligence Test (MSCEIT) Managing Emotions Score (3, 27, 31)
Social Skills	Social Skills Questionnaire (31) Social Skills Performance Assessment (37) Social Communication Questionnaire - Social Subscale (34)

### Data analysis

Separate meta-analyses were conducted for each psychometric outcome dimension. When studies used more than one measure to evaluate the same social cognition dimension, the measure that was most frequently used in all studies was selected. When studies used psychometric measures that were not used in any other study, the measure that most approximated the measure used in the majority of the remaining studies, based on the provided task description, was selected by consensus, after reviewing the available literature on the psychometric properties of the instrument in question with regards to convergent validity with the most frequently used task.

Extracted data was inputted into Meta-Essentials Workbook for Meta-Analysis (38) for differences between independent groups–continuous data (Version 1.3). This workbook computes bias-adjusted standardized mean differences (Hedges' g, expressed as 95% confidence intervals−95% CI), as well as combined effect sizes with hypothesis testing. We used a random-effects model for the meta-analyses. Positive effect sizes indicate a better performance by the schizophrenia groups compared to ASD groups. Individual studies were weighed according to the inverse variance weighting method, with an added between-studies variance component based on the DerSimonian-Laird estimator (39). Confidence intervals were estimated using the weighted variance method, as described previously (39). This approach takes into account the uncertainty resulting from the need to estimate heterogeneity variance and within-study variances, resulting in wider estimated confidence intervals for the combined effect size in analyses based on small numbers of studies. In the latter situation, and especially when heterogeneity is high, confidence intervals may include 0 even when classical z-distribution confidence intervals would not. To assess heterogeneity of studies, in each meta-analysis we used Cochran's Q test to examine the null hypothesis that all studies estimated the same effect. We further computed I^2^ to estimate the ratio of true heterogeneity to total observed variation, and Tau^2^ (T^2^) to estimate between-study variance (40). Publication bias was examined by means of funnel-plots, with Egger regression and trim-and-fill analysis for estimation of the adjusted effect size and of missing studies (41). Because schizophrenia and ASD have different ages of onset and different developmental and clinical courses, we evaluated the moderator effect of age on the meta-analyses, again using the resources provided by Meta-essentials, which, in essence, perform a weighted regression of the studies' effect sizes over the chosen continuous moderator variable, in this case participants' mean age (38).

## Results

### Study selection and characteristics

**Figure 1:**
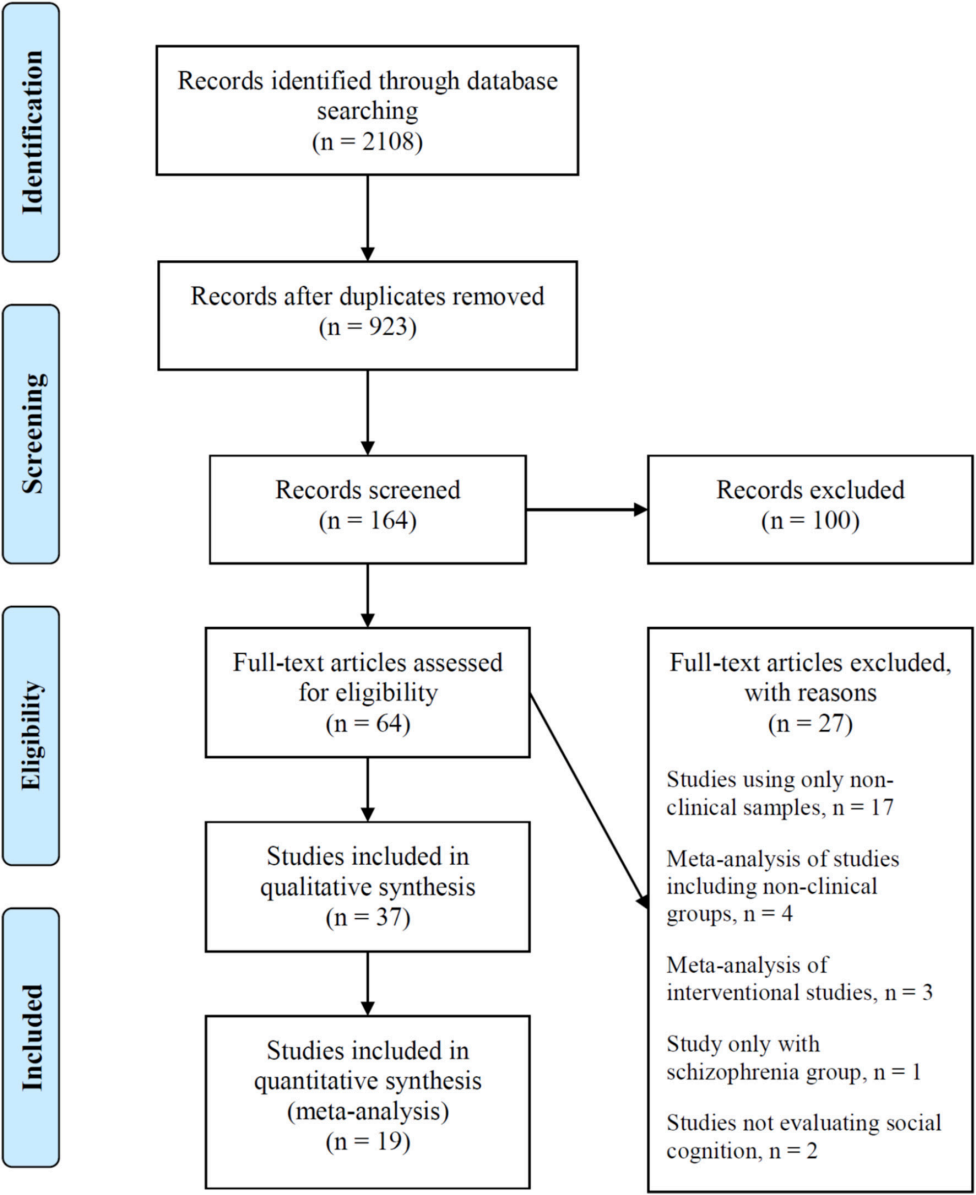
Flow diagram of included studies.

We identified 19 studies eligible for meta-analysis (Figure 1) (3, 10, 20, 22–37). The characteristics of these studies are presented in Table 2. Overall, 1,040 patients were included in the analyses (558 patients with schizophrenia and 482 patients with ASD). All but one study Murphy (10) included patients of both genders, although samples were predominantly constituted by male patients, particularly in the ASD groups. Studies were conducted in adolescent or adult populations; in 8 of the eligible studies, patients with schizophrenia were significantly older than patients with ASD [Craig et al. (30), Couture et al. (29), Eack et al. (3), Kandalaft et al. (27), Krawczyk et al. (31), Radeloff et al. (33), Sasson et al. (24), and Solomon et al. (34)]. Except for four studies that reported significantly higher mean IQ in ASD patients [Eack et al. (3), Kandalaft et al. (27), Murphy (10), and Solomon et al. (34)], no significant differences were found in mean IQ between patients with ASD and patients with schizophrenia.

**Table 2:** Characteristics of studies included in the meta-analysis.

**Study**	**Diagnosis and number of participants**		**Age, years mean (SD)**		**Gender male/female**		**IQ mean (SD)**	
	**SCZ (n)**	**ASD (n)**	**SCZ**	**ASD**	**SCZ**	**ASD**	**SCZ**	**ASD**
Bolte et al. ^*^ (26)	Schizophrenia (21)	Autism (35)	Simplex: 17.6 (3.1) Multiplex: 20.0 (2.8)	Simplex: 15.7 (8.6) Multiplex: 12.1 (3.2)	Simp: 12/5 Multi: 3/1	Simp: 12/3 Multi: 17/3	Non-verbal; Simplex: 100.2 (12.9) Multiplex: 107.5 (4.9)	Non-verbal; Simplex: 103.7 (23.7) Multiplex: 95.4 (25.0)
Couture et al. 2010 (29)	Schizophrenia (44)	Autism (36)	**27.5** **(6.3)**	**20.9** **(5.7)**	39/5	29/7	98.9 (15.8)	101.3 (17.8)
Craig et al. (30)	Deluded schizophrenia and delusional disorder (16)	Asperger's syndrome (17)	**31.69** **(9.85)**	**24.12** **(6.72)**	11/5	15/2	105.14 (8.42)	104.76 (7.11)
Eack et al. (3)	Schizophrenia; Schizoaffective disorder (47)	Autism spectrum disorders (43)	**34.96** **(12.4)**	**24.86** **(5.75)**	34/13	38/5	**99.04** **(10.50)**	**113.05** **(15.28)**
Kandalaft et al. (27)	Schizophrenia (18)	Asperger's syndrome (20)	**31.05** **(5.42)**	**22.85** **(4.9)**	12/7	16/4	**102.94** **(12.8)**	**113.22** **(11.09)**
Krawczyk et al. (31)	Schizophrenia; Schizoaffective disorder (13)	Autism spectrum disorders without language delay (15)	**30.0** **(5.72)**	**21.73** **(4.39)**	7/6	11/4	101.67 (12.84)	107.5 (14.07)
Lugnegård et al. (32)	Schizophrenia; Schizoaffective disorder; Schizophreniform disorder (36)	Asperger's syndrome (53)	28.8 (4.1)	27.3 (4.1)	22/14	26/27	Verbal; 9.9 (2.1)	Verbal; 10.4 (2.3)
Martinez et al. (19)	Schizophrenia (36)	Autism spectrum disorders (19)	23.4 (3.5)	22.7 (4.1)	30/6	15/4	101.2 (13.5)	108.6 (16.9)
Morrison et al. (37)	Schizophrenia (54)	Autism spectrum disorders (54)	28.67 (10.11)	25.67 (7.17)	47/7	47/7	103.32 (10.98)	106.02 (12.83)
Murphy (9)	Schizophrenia with delusions and/or auditory hallucinations detained under Mental Health Act (13)	Asperger's syndrome detained under Mental Health Act (13)	29.7 (6.2)	35 (7.5)	All male		**82.2** **(9.6)**	**102** **(15.5)**
Radeloff et al. (33)	Schizophrenia (21)	Asperger's syndrome; Childhood autism; Atypical autism (34)	**24.67** **(5.20)**	**19.06** **(5.12)**	16/5	31/3	103.33 (11.21)	105.73 (12.92)
Sachse et al. (28)	Paranoid schizophrenia (19)	High-functioning autism spectrum disorders (22)	25.5 (4.9)	20.9 (5.6)	14/5	18/4	100.1 (12.0)	100.1 (13.3)
Sasson et al. (23)	Schizophrenia with minimal symptoms (10)	Autism (10)	28.1 (5.07)	23.0 (5.27)	9/1	All male	98.5 (12.99)	107.8 (17.15)
Sasson et al. (24)	Schizophrenia (44)	Autism spectrum disorders (21)	**35.34** **(10.56)**	**23.43** **(4.36)**	27/17	18/3	94.11 (20.28)	101.48 (16.97)
Solomon et al. (34)	First-episode psychosis including schizophrenia, schizoaffective disorder, schizophreniform disorder and psychosis NOS (16)	Autism; Asperger's syndrome (20)	**17.06 (1.81)**	**15.15 (2.28)**	12/4	16/4	**98.69 (15.45)**	**106.1 (15.39)**
Tin et al. (35)	Clinically stable schizophrenia (30)	High-functioning autism (30)	17.47 (1.22)	17.03 (0.93)	19/11	23/7	113.03 (9.61)	109.93 (12.53)
Tobe et al. (22)	Schizophrenia; Schizoaffective disorder (92)	High-functioning autism spectrum disorders (19)	36.0 (11.8)	39.4 (12.5)	79/13	17/2	Not reported	Not reported
Vírseda Antoranz et al. (36)	Schizophrenia (18)	Asperger's syndrome (6)	29.55 (5.3)	25.5 (4.96)	10/8	All male	100.88 (15.49)	95.16 (22.17)
Waris et al. (25)	Schizophrenia (10)	Pervasive developmental disorders (15)	16.2 (1.6)	16.1 (1.6)	2/8	7/8	92 (18.8)	96.9 (12.6)

Bold values indicate significant differences as reported in the original articles.

* *Data on age and intelligence quotient (IQ) presented separately for families with one (simplex) or more than one (multiplex) child affected by the disorder; no statistics were reported for differences in age or IQ between simplex and multiplex samples or groups. ASD, autism spectrum disorders; NOS, not otherwise specified; SCZ, schizophrenia; SD, standard deviation*.

Eighteen additional studies were not eligible for meta-analysis (42–59). These included 10 functional or morphometric imaging studies that did not provide adequate data for quantitative methods [Chen et al. (42), Ciaramidaro et al. (43), Eack et al. (45), Hirata et al. (46), Katz et al. (47), Mitelman et al. (49), Parellada et al. (52), Pinkham et al. (54), Serrano et al. (57), and Stanfield et al. (58)], and 8 additional studies that presented data that was either considered ineligible for inclusion in meta-analyses or evaluated social cognition dimensions that were not investigated in more than 3 independent studies: Corbera et al. (44), Le Gall et al. (48), Ozguven et al. (50) and Pomarol-Clotet et al. (56) presented only qualitative data in abstract form; Ozguven et al. (51) presented only data as minimum-maximum and median values and used non-parametric test statistics that were not suitable for inclusion in the meta-analysis; Pilowsky et al. (53) evaluated false beliefs and deception, and Pinkham et al. (55) evaluated paranoia; finally, Van Lancker et al. (59) only presented auditory emotion perception data, and separately for younger and older children with autism. These studies are all summarized in Table 3 and will not be further analyzed here.

**Table 3:** Characteristics of studies that were not included in the meta-analyses.

**Study**	**Diagnosis and number of participants**		**Age, years mean (SD)**		**Gender male/female**		**Summary of main findings**
	**SCZ (n)**	**ASD (n)**	**SCZ**	**ASD**	**SCZ**	**ASD**	
Chen et al. 2017 (42)	Schizophrenia (35)	Autism spectrum disorders (22)	15.6 (1.8)	13.1 (3.1)	20/15	15/7	fMRI study. Shared atypical brain connections were mostly found in the SN and DMN. In ASD, the distinct atypical connectivity was mainly intra-SN connections, while in SCZ it was mainly inter-DMN-SN connections. Shared atypical DMN and SN connections in ASD were significantly related to social deficits; no significant relationship between the connections and the PANSS scores was observed in the SCZ group.
Ciaramidaro et al. 2015 (43)	Paranoid schizophrenia (18)	Autism (23)	14-32 (min-max)	13-33 (min-max)	18/4	23/2	fMRI study. ASD group committed more errors than SCZ group in a ToM task, while the latter showed higher reaction times. Increased activation for physical information processing in SCZ, and decreased activation for intentional information processing in ASD. Increased connectivity patterns between the right PSTS and VMPFC in SCZ but decreased in ASD.
Corbera et al. 2017 (44) (abstract)	Schizophrenia (49)	Autism spectrum disorders (31)	NA	NA	NA	NA	Greater deficits in empathy for emotional pain in ASD vs. NTC than SCZ vs. NTC. Deficits in perspective taking and personal distress were present in both SCZ and ASD, while deficits on empathic concern and overall empathy were present in ASD only.
Eack et al. 2017 (45)	Schizophrenia (36)	Autism spectrum disorders (36)	26.25 (6.83)	23.91 (6.05)	22/14	30/3	fMRI study. SCZ performed significantly better than ASD in simple perspective taking trials while both groups performed similarly but significantly worse than NTC in complex trials. SCZ with significantly greater VMPFC and left TPJ activity than ASD. Dense connections between MPF circuit and TPJ in ASD, while only connections between DL and VMPFC in SCZ. Increased bilateral orbitofrontal connectivity in ASD compared to SCZ.
Hirata et al. 2018 (46)	Schizophrenia (15)	Autism spectrum disorders (13)	36 (29-47)^*^	30 (23.3-38.5)^*^	12/3	12/1	fNIRS study. No difference between ASD and SCZ in a facial emotion recognition task. Left frontotemporal area dysfunction during non-social and social cognition tasks in ASD, which was associated with ‘exaggerated attention’. Frontopolar area dysfunction for non-social cognition task in SCZ, which was associated with severity of symptoms.
Katz et al. 2016 (47)	Schizophrenia (24)	High functioning autism (23)	31.21 (8.21)	26.65 (6.51)	All Male	All Male	Whole-brain tractography and VBM study. SCZ and HFA shared common long-range white matter deficits but opposite gray matter abnormalities (decreased volumes in SCZ and increased volumes in ASD).
Le Gall et al. 2012 (48) (abstract)	Early-onset schizophrenia (24)	High functioning autism (14); Asperger's syndrome (18)	13.4 (0.57)	HFA: 12.3 (0.74) AS: 12.3 (0.65)	NA	NA	No difference in attribution of intentions ability was observed between SCZ, HFA and AS. HFA showed increased difficulty in understanding figurative language compared to SCZ and AS. HFA/AS had more severe pragmatic impairments than SCZ, as evaluated by the Children's Communication Checklist.
Mitelman et al. 2017 (49)	Schizophrenia (49)	Autism spectrum disorders (20)	42.7 (12.3)	28.4 (6.5)	42/7	17/3	MRI morphometric study. Participants with SCZ showed a pattern of decreased gray matter and increased white matter volumes compared to participants with ASD, particularly in motor-premotor and anterior frontal cortex, anterior cingulate, fusiform, superior and middle temporal gyri.
Ozguven et al. 2007 (50) (abstract)	Schizophrenia (20)	Asperger's syndrome (16)	Range 18-37	Range 18-37	All Male	All Male	ToM was more impaired in participants with AS compared to those with SCZ. Second-order ToM performance was correlated with negative but not paranoid symptoms in SCZ.
Ozguven et al. 2010 (51)	Schizophrenia (20)	Asperger's syndrome (14)	27.4 (4.75)	24.4 (7.1)	All Male	All Male	Participants with AS performed significantly worse than SCZ in first-order ToM items, while both groups performed significantly worse than NTC in second-order ToM items.
							In ASD there was a positive correlation between first order ToM and verbal comprehension, while in SCZ a similar correlation was found with second-order ToM, in addition to a negative correlation of this latter measure with negative symptom scores.
Parellada et al. 2017 (52)	Early-onset first episode psychosis (29)	Autism spectrum disorders (30)	14.1 (0.98)	13.3 (1.99)	18/11	28/2	Region-of-interest and VBM study. Gray matter volume reductions in the right anterior insula and bilateral posterior insula were present both in ASD and FEP. Regional insular volume deficits were associated with severity of symptoms (social communication and insight deficits) in both ASD and FEP.
Pilowsky et al. 2000 (53)	Childhood-onset schizophrenia (12)	High functioning autism (12)	12.2 (1.7)	13.0 (3.9)	9/3	11/1	No differences between groups in fact and value belief tasks and a false belief task. Individuals with SCZ performed significantly better than those with HFA in a deception task. In HFA, ToM ability was correlated with verbal ability.
Pinkham et al. 2008 (54)	Paranoid and non-paranoid schizophrenia/ schizoaffective disorder (12 P, 12 NP)	High functioning autism spectrum disorders (12)	P: 26.42 (5.25) NP: 28.0 (3.93)	24.08 (5.71)	All Male	All Male	fMRI study. No significant differences were found between the groups in a trustworthiness task except for a better performance of non-paranoid vs. paranoid schizophrenia patients. P-SCZ ans ASD with reduced left VLPFC activation compared to NP-SCZ during the trustworthiness task.
Pinkham et al. 2012 (55)	Paranoid and non-paranoid schizophrenia/ schizoaffective disorder (24 P, 30 NP)	Autism spectrum disorders (18)	P: 27.33 (5.96) NP: 29.87 (7.18)	24.56 (6.0)	P: 21/3 NP: 25/5	17/1	Overall similar level of paranoia in SCZ and ASD groups. Paranoia in SCZ associated with victimization, suspicion, and threat of harm (suggesting externalizing bias), while in ASD it was associated with social cynicism (suggesting increased impairment in understanding social cues and rules of social interaction).
Pomarol-Clotet et al. 2005 (56) (abstract)	Schizophrenia (33)	Asperger's syndrome (24)	NA	NA	NA	NA	No difference in performance in ToM tasks between groups. Executive function and memory impairments only present in the SCZ group.
Serrano et al. 2014 (57) (abstract)	First episode psychosis patients (29)	Autism spectrum disorders (30)	13.33 (1.99)	13.08 (2.43)	NA	NA	VBM study. FEP patients had smaller right hemisphere cingulate isthmus volume and cortical thickness than patients with ASD.
Stanfield et al. 2017^a^ (58)	Schizotypal personality disorder (21)	Autism spectrum disorders (28)	37.1 (9.2)	39.5 (11.6)	14/7	22/6	fMRI study. No significant differences in an emotion recognition task (Ekman 60) and Social Judgements task between SCZ and SPD. SPD group showed significantly greater activation compared to ASD group when making social judgements compared to gender judgements in the amygdala and 3 clusters: right posterior cerebellum, extending into the fusiform and inferior temporal gyri; left posterior cerebellum; and left intraparietal sulcus extending through the medial portions of the temporal gyri into the fusiform gyrus.
Van Lancker et al. 1989 (59)	Schizophrenia (19)	Autism (28)	9.6 (2.1)	Younger: 6.9 (1.2) Older: 11.3 (3.1)	NA	NA	SCZ group performed significantly better on an auditory emotion recognition task compared to the ASD groups (both younger and older participants).

AS, Asperger syndrome; ASD, Autism Spectrum Disorders; DMN, Default Mode Network; ER, emotion recognition; FEP, First Episode Psychosis; fMRI, functional Magnetic Ressonance Imaging; fNIRS, functional near-infrared spectroscopy; HFA, High Functioning Autism; MRI, Magnetic Ressonance Imaging; NA, not available; NP, Non-paranoid; NTC, neurotypical controls; P, paranoid; PANSS, Positive and Negative Syndrome Scale; PSTS, posterior superior temporal sulcus; SD, Standard deviation; SPD, Schizotypal Personality Disorder; SN, Salience Network; SCZ, Schizophrenia; ToM, theory of mind; VBM, voxel based morphometry; VLPFC, ventrolateral prefrontral cortex; VMPFC, ventromedial prefrontal cortex.

* Results presented as medians and interquartile ranges.

a *This study used a sample of subjects with Schizotypal Personality Disorder*.

### Synthesized findings

#### Emotion perception

Eight studies provided data on emotion perception (3, 22–28). Two studies used the Penn Emotion Recognition Task (ER-40) (3, 22), 2 other studies used facial affect recognition tests based on photographs by Ekman & Friesen (26, 27) and the remaining four studies (23–25, 28) each used different, less commonly used instruments, although all of them designed to evaluate the correct identification of facial affect from images of human faces. The study by Couture et al. (29) was excluded from the emotion perception meta-analysis because it did not report total scores on the Movie Stills Task with Faces, but only the individual sub-scores for a limited selection of emotions (sad, afraid, and angry) (29). The study by Tin et al. was excluded from this meta-analysis because it used a computerized task with cartoons where affective inferences were made based on verbal and eye gaze cues and not facial affect expression (35).

**Figure 2:**
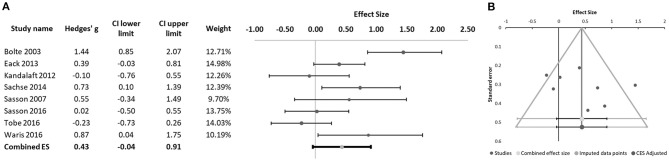
**(A)** Forest plot of studies evaluating emotion perception. Dots represent each study, with dot size reflecting study weight in the model. Error bars indicate the effect size (with confidence interval) of each study. Bottom line represents the combined effect size with its confidence interval. **(B)** Funnel plot of studies evaluating emotion perception.

**Figure 3:**
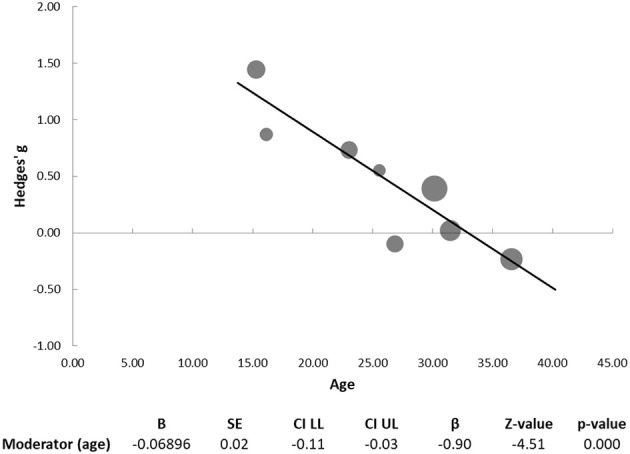
Regression of age on effect size for emotion perception studies. Dots represent each study, with dot size reflecting study weight in the model. The graph plots the effect-size of each study against the corresponding value of the moderator. Statistics for moderator analysis are presented in the bottom table.

We found a significant difference between schizophrenia and ASD patients in emotion perception, with the schizophrenia group performing better than the ASD in these tasks (Hedges' g = 0.43, 95% CI −0.04 to 0.91; *p* = 0.031; Figure 2A). We found significant heterogeneity of effect sizes according to the Q-test (Q = 25.00; *p* = 0.001), with an I^2^ value of 72%. No missing studies were identified in the trim and fill analysis. Funnel plot analysis did not reveal marked asymmetry (Figure 2B) and Egger's regression did not suggest publication bias (intercept = 4.82, 95% CI: −7.32 to 16.96; *t* = 0.94, *p* = 0.384). Participants' mean age was found to have a significant moderator effect (B = −0.069; *p* < 0.001), with larger effect sizes for between-group differences (favoring better performance in the schizophrenia groups) observed in studies with younger participants (Figure 3). Some of the studies included in the meta-analysis provided additional information regarding differences between individuals with schizophrenia and ASD in particular aspects of the emotion recognition process. Sachse et al. (28) compared emotion perception in 19 participants with paranoid schizophrenia and 22 participants with high-functioning ASD using a combination of visual form discrimination and facial processing tasks (the Benton Visual Form Discrimination Test and the Benton Facial Recognition Test, respectively), and a facial emotion recognition task (the Frankfurt Test for the Recognition of Facial Affects). Individuals with schizophrenia showed reduced visual perception capacity (namely, more marked difficulties in visual form discrimination) while individuals with ASD had poorer facial identity recognition and poorer facial emotion recognition, particularly for complex emotions, suggesting that different cognitive processes may underlie emotion recognition difficulties in these two disorders (28). In the study by Sasson et al. (23), although the schizophrenia (*n* = 10) and ASD (*n* = 10) groups did not differ in emotion perception performance in a social scenes task where faces expressing a single emotion were either present or digitally erased, differences were found when eye tracking data were analyzed: individuals with schizophrenia oriented gaze to face regions more rapidly when faces were present relative to stimuli from which faces had been removed, while the autism groups oriented gaze to the face region at the same speed regardless of whether the face was present or not (23). In a later study, Sasson et al. (24) again found no significant differences between the schizophrenia (*n* = 44) and ASD (*n* = 21) groups in emotion recognition accuracy. The two clinical groups only differed from the neurotypical control group when faces were presented integrated into congruent and incongruent emotional contexts, but not when faces were presented in isolation. Interestingly, while patients with schizophrenia and neurotypical participants showed increased fixation time to the face region when faces were presented within an incongruent emotional context compared to when they were integrated into a congruent emotional context, this was not observed in the ASD group, who spent the same time fixating the face region regardless of emotional context congruency. Moreover, in individuals with schizophrenia, emotion recognition accuracy correlated with IQ, while this was not the case in individuals with ASD (24). Finally, Tobe et al. (22) used an emotion perception paradigm comprising an auditory emotion recognition battery (audio recordings of sentences with neutral content that were read using different emotional tones) and a visual emotion recognition battery (ER-40). While participants with schizophrenia (*n* = 92) were impaired in both auditory and visual tasks, participants with high-functioning ASD (*n* = 19) were impaired only in the visual emotion recognition task (22).

#### Theory of mind

**Figure 4:**
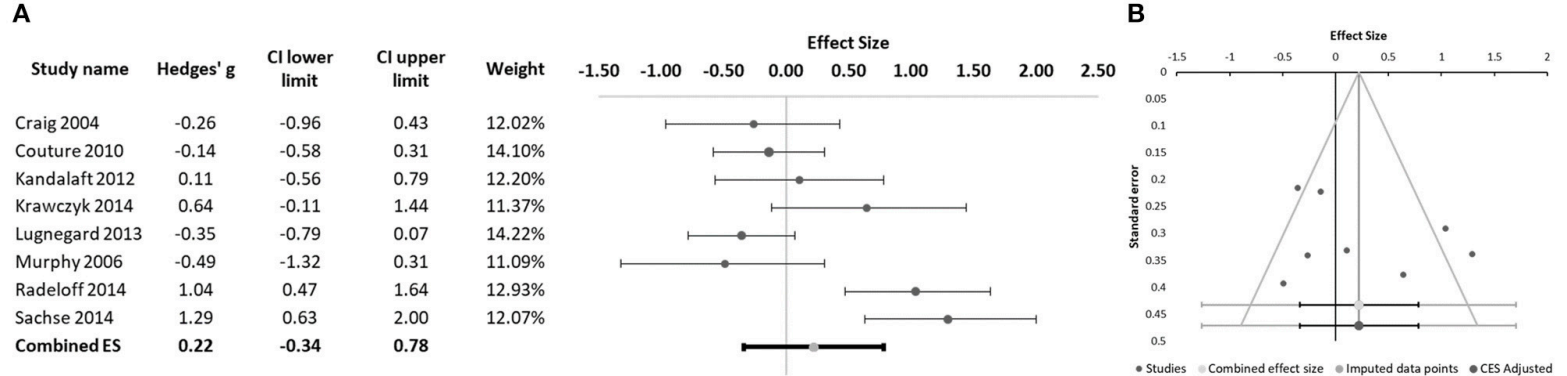
**(A)** Forest plot of studies using the Reading the Mind in the Eyes Test (RMET). Dots represent each study, with dot size reflecting study weight in the model. Error bars indicate the effect size (with confidence interval) of each study. Bottom line represents the combined effect size with its confidence interval. **(B)** Funnel plot of studies using the RMET.

**Figure 5:**
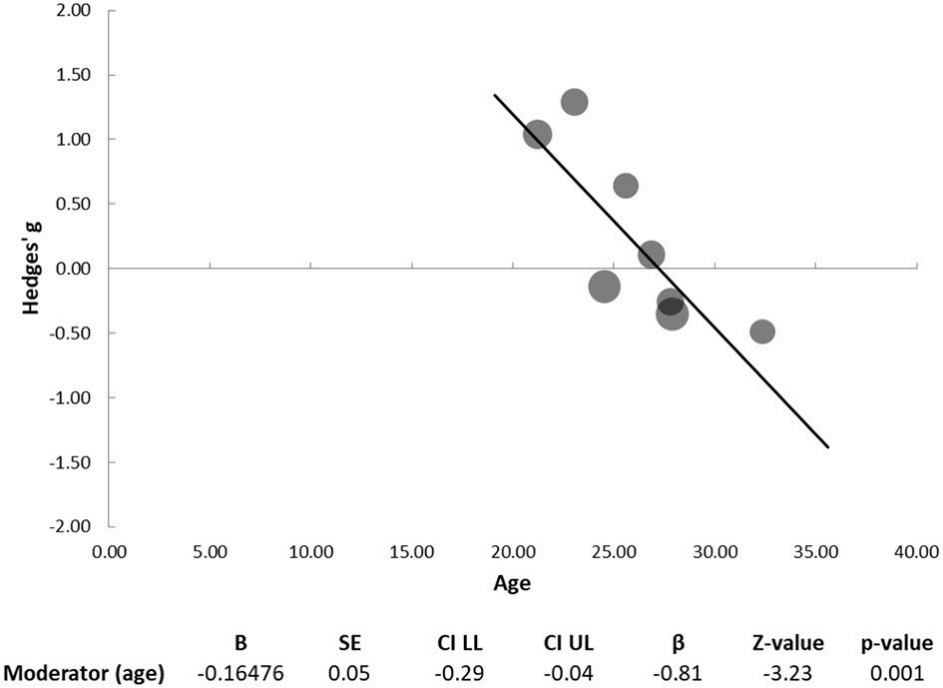
Regression of age on effect size—studies using the Reading the Mind in the Eyes Test. Dots represent each study, with dot size reflecting study weight in the model. The graph plots the effect-size of each study against the corresponding value of the moderator. Statistics for moderator analysis are presented in the bottom table.

Because literature is contradictory regarding the dimension of social cognition that is assessed by the RMET, with several authors considering this test a measure of mentalizing capacity (29, 60) and others considering it a measure of emotion recognition rather than of ToM ability (61), we chose to separately analyze the 8 studies that used the RMET. The meta-analysis of these 8 studies (10, 27–33) showed no significant differences in performance between the schizophrenia and ASD groups (Hedges' g = 0.22, 95% CI −0.34 to 0.78; *p* = 0.351; Figure 4A). The Q-test was significant (33.66; *p* < 0.001) and I^2^ was 79.20%. Funnel plot analysis did not reveal marked asymmetry (Figure 4B) and Egger's regression was not significant (intercept = 3.85; 95% CI: −17.14 to 24.84; *p* = 0.680). Age was found to have a significant moderator effect (B = -0.165; *p* = 0.001), with larger effect sizes (favoring a better performance by patients with schizophrenia) in studies with younger participants (Figure 5).

**Figure 6:**
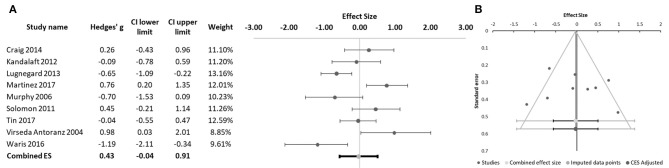
**(A)** Forest plot of studies evaluating theory of mind (inferencing). Dots represent each study, with dot size reflecting study weight in the model. Error bars indicate the effect size (with confidence interval) of each study. Bottom line represents the combined effect size with its confidence interval. **(B)** Funnel plot of studies evaluating theory of mind (inferencing).

Data on mental state inference was obtained from 9 studies (10, 19, 25, 27, 30, 32, 34–36). The tasks that were used varied significantly between studies. Two studies [Kandalaft et al. (27) and Lugnegård et al. (32)] used the Triangles Animation Task, while the remaining studies each used a different measure of ToM. All ToM measures evaluated inferences about intentions or beliefs. When data was presented separately for first order Tom (inference about what a character is thinking) and for second order ToM (inference about what a character thinks another character is thinking), only second order scores were considered for meta-analysis as these better resemble the type of attributions evaluated by the measures used in the other studies. No significant difference was found between schizophrenia and ASD patients in ToM performance (Hedges' g = −0.03, 95% CI −0.56 to 0.50; *p* = 0.903; Figure 6A). Heterogeneity was significant with a Q-test of 33.0 (*p* < 0.001), and an I^2^ value of 75.76%. The funnel plot was symmetrical (Figure 6B), with no missing studies identified in the trim and fill analysis. Egger's regression did not suggest significant publication bias (intercept = 1.0, 95% CI: −13.53 to 15.53; *t* = 0.16, *p* = 0.878). Age of participants did not have a moderator effect on mental state inference ability (*p* = 0.993). Several studies included in this meta-analysis provided additional relevant information regarding specific aspects of ToM performance. Martinez et al. (19) found that participants with schizophrenia (*n* = 36) had a better performance than individuals with ASD (*n* = 19) in attribution of mental states using the Movie for the Assessment of Social Cognition (MASC) test, that assesses subtle inference abilities. Both groups performed significantly worse than neurotypical controls in the over-mentalizing measure of the MASC, showing a high number of wrong answers on the task that reflects overly complex mental state reasoning (62). However, only the ASD group performed significantly worse than controls in the under-mentalizing and the no-mentalizing measures, that indicate overly simplistic or complete lack of mental state inference capacity, respectively (62). Moreover, the ToM score was negatively correlated with the Positive and Negative Syndrome Scale (PANSS) disorganization score in the schizophrenia group and with the Autism Quotient score in both clinical groups (19). Tin et al. used a Faux Pas Task to evaluate ToM in 30 individuals with schizophrenia and 30 individuals with high-functioning autism, and found that subjects with autism performed significantly worse than schizophrenia patients in the *Faux Pas* measures of recognition, understanding, and inference of emotion, but not inference of intention, a dimension for which groups performed equally (35). Craig et al. found a negative correlation between the Hinting Task Score (a ToM loading task) and scores in the Paranoia Scale (*r* = −0.25, *p* < 0.05), suggesting that high levels of paranoia symptoms are associated with heavier compromise of ToM ability (30). Lugnegård et al. (32) was the only study addressing the issue of gender effects on ToM ability in both ASD and schizophrenia, and, using the Triangles Animation Task, found that men with schizophrenia (*n* = 22) perform worse than men with Asperger's syndrome (*n* = 26) in the Intentionality score (ability to describe complex, intentional mental states), while no such difference was observed in females. In contrast, women with schizophrenia (*n* = 14) performed worse than women with Asperger's syndrome (*n* = 27) in the Appropriateness Score (capacity to adequately describe the actions in an animation), with no differences between men of both groups in this measure (32).

#### Emotional intelligence and social skills

**Figure 7:**
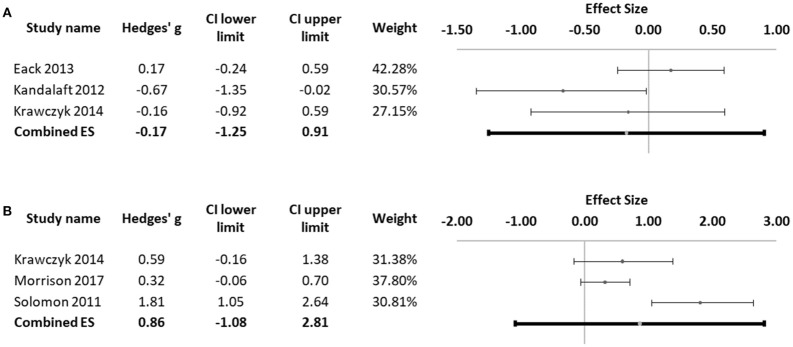
**(A)** Forest plot of studies comparing scores in the managing emotions score of the MSCEIT. **(B)** Forest plot of studies evaluating social skills. Dots represent each study, with dot size reflecting study weight in the model. Error bars indicate the effect size (with confidence interval) of each study. Bottom line represents the combined effect size with its confidence interval.

Three studies assessed emotional intelligence (3, 27, 31) using the Mayer–Salovey–Caruso Emotional Intelligence Test (MSCEIT). We conducted a meta-analysis of the Managing Emotions Score of the MSCEIT, as this is included as the measure of social cognition in the MATRICS Consensus Cognitive Battery for schizophrenia (63). No significant difference was found between schizophrenia and ASD patients in this measure of emotional intelligence (Hedges' g = −0.17, 95% CI −1.25 to 0.91; *p* = 0.490; Figure 7A). The Q-test was not significant (Q = 4.75; *p* = 0.093), with an I^2^ value of 57.88%. Funnel plot and Egger statistic were not interpretable due to the low number of studies (data not shown).

Social skills were evaluated in 3 of the eligible studies (31, 34, 37). Each of these studies used a different scale to evaluate social skills: Krawczyk et al. (31) used the Social Skills Questionnaire, Solomon et al. (34) used the Social Subscale of the Social Communication Questionnaire and Morrison et al. (37) used the Social Skills Performance Assessment (Table 1). No significant difference between the two groups was found in this domain, despite a trend for patients with schizophrenia to perform better than subjects with ASD (Hedges' g = 0.86, 95% CI −1.08 to 2.81; *p* = 0.056; Figure 7B). Marked heterogeneity was observed with an I^2^ value of 82.84% and a highly significant Q-test value of 11.66 (*p* = 0.003), suggesting poor comparability between the different social skills measures used in the original studies. Funnel plot and Egger statistic were not interpretable due to the low number of studies (data not shown). In the study by Morrison et al. (37), participants with schizophrenia (*n* = 54) showed significantly less repetitive movements and asked significantly more questions than participants with ASD [*n* = 54] who, in turn, scored better on clarity and flat affect (37). Based on this finding, the authors suggest that while a pattern of inappropriate nonverbal behavior with more frequent social interactions is characteristic of schizophrenia, ASD display a pattern of inappropriate verbal content and poorer interactive behavior (37). Finally, social skills were found to correlate significantly with IQ in the schizophrenia group but not in the ASD group (37). A similar finding was reported by Solomon et al. who also found more repetitive behaviors and worse scores on the social domain in ASD patients (*n* = 20) compared to patients with first-episode psychosis, while the latter showed higher scores in the Awareness (cognizance of social cues) and Communication (interpersonal expressiveness and conversational give-and-take) subscales of the Social Responsiveness Scale (34).

### Risk of bias

Studies included in the meta-analyzes were characterized by low sample sizes (mean sample size for schizophrenia groups was 29.4 participants and for ASD groups was 25.4 participants). Additional sources of potential selection bias include the following: (1) diagnostic variability, with some studies including more broadly-defined psychotic syndromes and ASD; (2) differences in mean age across diagnostic groups, participants with schizophrenia being significantly older in 8 of the 19 studies; (3) differences in IQ across the two clinical groups, with higher IQs in ASD participants in 4 studies.

Another frequent limitation found in studies included in the meta-analyses concerns the absence of measures to reduce measurement bias. Most studies do not mention if raters were adequately trained in the application of social cognitive tasks or if they were blinded to the participants' diagnosis. Notable exceptions were: (1) Craig et al. who used a second blinded rater in the coding of a sample of transcripts of the Attributional Style Structured Interview (ASSI) (29); (2) Eack et al. who explicitly mention that raters were trained in social cognition measures and supervised by an experimented psychologist (3); (3) Lugnegård et al. who blinded raters in the scoring procedure of the Triangles Animation Task (32); and (4) Morrison et al. who trained two raters to improve reliability at study-begin, with re-assessment of inter-rater reliability at mid-point and at study end, in addition to ensuring that raters were blinded to subjects' diagnosis (37).

## Discussion

### Summary of main findings

The need for direct comparisons of social cognitive performance between patients with schizophrenia and ASD is amply recognized in the literature as a fundamental contribution to a better understanding of the similarities and differences between these two neurodevelopmental disorders (1). Here we systematically reviewed the available literature reporting direct head-to-head comparisons between individuals with schizophrenia and subjects with ASD in terms of social cognitive performance, and performed separate meta-analyses of the results regarding various dimensions of social cognition. We found 38 studies reporting comparisons of social cognitive performance in schizophrenia and ASD. Nineteen of these studies were eligible for meta-analyses. Eight of these studies compared emotional perception across the two groups. Mentalizing capacity, as assessed by the RMET, was evaluated in eight studies, while a further nine studies compared mental state inference capacity in the two groups. Emotional intelligence and social skills were each studied in three independent studies, and a number of isolated studies addressed other, less studied social cognition dimensions and skills.

The main finding from our meta-analysis was that ASD subjects are significantly more impaired than patients with schizophrenia in emotion perception from faces, with a combined medium effect size of 0.43 (*p* = 0.003). Furthermore, we found that age significantly influences the effect size of this difference in performance, so that with increasing age the difference in emotion perception ability between ASD subjects and patients with schizophrenia disappears. This may reflect, on the one hand, a deterioration of social cognitive skills in schizophrenia patients with increasing illness duration, and on the other hand an age-dependent improvement of emotion perception skills in ASD, probably as a result of social learning and accumulating social experience. Indeed, Lever and Geurts found that ToM deficits observed throughout adulthood in ASD were no longer present in older (50+ years) adults (64), and Magiati et al. (65), in a review of 25 studies that looked at the longitudinal evolution of cognitive, linguistic, social and behavioral outcomes in patients with ASD, found evidence of significant (albeit not always consistent) improvement in all these domains, and specially so in communication skills and adaptive functioning (65). Given that only approximately 25% of patients with schizophrenia have a poor long-term outcome (66) and that cognitive and social deficits, although present early in the disease evolution, do not appear to deteriorate over time (1, 66, 67), the main factors driving the dissipation of group-differences with increasing age are likely to reflect the well-known age-dependent improvement in ASD core symptoms that is characteristic of this disorder's natural evolution in adulthood. Surprisingly, the meta-analysis of studies that compared performance of participants with schizophrenia and ASD subjects on the RMET, while again finding a significant moderator effect of age, did not find significant differences between the two groups regarding performance of this specific task. This suggests that the RMET may tap into additional components of social cognition other than basic emotion recognition or that it may be more sensitive to factors like verbal IQ, that was often lower in participants with schizophrenia compared to those with ASD. Notwithstanding this, the fact that age only moderated effect sizes on emotion perception and RMET, but not other ToM tasks, suggests that emotion perception is a significant dimension of the type of mentalizing capacity assessed by the RMET. Oakley et al. argue that the RMET may in fact measure emotion recognition rather than ToM ability, based on the observation that patients with ASD and neurotypical controls matched for alexithymia scores do not differ in RMET performance but do so on inference ability measured by the MASC (61).

Other relevant findings from studies comparing emotion perception in ASD and schizophrenia include a tendency for lower relevance of emotional context when judging facial emotions in the ASD groups compared to patients with schizophrenia (23, 24). This is in line with previous findings that patients with ASD have a diminished orientation to social stimuli, which in turn is believed to contribute critically to the impaired social cognitive ability typical of the disorder (68). By contrast, deficits in emotion perception in schizophrenia are much more dependent on general cognitive ability (24, 31, 58). Krawczyk et al. (31), for instance, found a significant positive correlation between emotion recognition capacity and analogical reasoning capacity in a group of patients with schizophrenia (*n* = 13) that was not present in a comparison group of subjects with ASD (*n* = 15) (31). Lysaker et al. also found a significant positive correlation between emotion recognition capacity and both education level and cognitive flexibility as assessed by the Wisconsin Card Sorting Test (69); finally, Mehta et al. showed that cognitive ability (particularly, the combination of cognitive flexibility and memory encoding ability) may explain up to 39% of variance in emotion recognition in schizophrenia (70).

Our meta-analysis found no differences between patients with schizophrenia and ASD in terms of mental state inference as measured by a variety of tasks and instruments. Where differences were found, they tended to favor a better performance by patients with schizophrenia compared to those with ASD (19, 35, 36), with the exception of a more pronounced attributional bias (favoring external attributions regarding negative events and personal external attributions) in patients with schizophrenia compared to those with ASD (30). The same applies to the findings of studies that were ineligible for inclusion in the meta-analyses. These studies report either no differences between the two disorders or a better performance by patients with schizophrenia (43, 45, 51, 53).

Specifically regarding schizophrenia, there seems to be converging evidence that mental state inference skills are critically influenced by the severity of clinical symptoms of this disorder, namely disorganization (19), paranoia scores (29, 30, 54) and negative symptoms (51). The same applies to cognitive deficits, that appear to have a more pronounced effect on social cognitive impairments in schizophrenia than they do in ASD, namely on such social cognitive dimensions as first- and second-order ToM, faux pas recognition, and social perception (1, 45, 70).

Meta-analyses of studies comparing social skills and emotional intelligence between ASD subjects and patients with schizophrenia also showed no difference between the two groups on these measures of social cognition, although, due to the low number of studies in each analysis, these were likely underpowered to find small or moderate effect sizes.

Together, the reviewed literature suggests that, other than in the ability to recognize emotions from perceived faces (a social cognitive dimension where ASD subjects are clearly more impaired than patients with schizophrenia) there seem to be no clear and consistent differences between ASD and schizophrenia in terms of social cognitive performance. There are at least three possible explanations for this: (1) ASD and schizophrenia are partly overlapping disorders with partly overlapping social cognition deficits and partly overlapping neurobiology; (2) social cognition deficits in ASD and schizophrenia are the final, common outcome of differing developmental pathways and neurobiological mechanisms; (3) the instruments that are in common use to assess social cognition in these two disorders lack the necessary specificity to discriminate between them, or at least are not sensitive enough to qualitative differences between the two disorders. It is likely that all three explanations are valid. Schizophrenia and ASD are two severely impairing neuropsychiatric disorders with partly overlapping genetic risk, and partly shared neurobiological abnormalities (18, 19). While such shared neurodevelopmental abnormalities could lead to similar social cognition deficits, functional neuroimaging studies do suggest that these deficits have partly diverging neural network correlates (43, 45, 58, 71).

Finally, many studies have found that despite being quantitatively similar, the social cognitive deficits found in ASD and schizophrenia are qualitatively distinct. For instance, social cognitive impairments in schizophrenia are heavily influenced by attributional bias in schizophrenia, while in ASD apparently similar social cognitive impairments predominantly correlate with a hypomentalization bias, where social stimuli and information are given lower relevance for making social judgements (68).

The importance of exploring differences and similarities in social cognition between schizophrenia and ASD has more than just theoretical implications. A better understanding of the mechanisms that underlie and differentiate social cognitive impairments in the two disorders will help develop disorder-tailored interventions that are capable of improving social functioning. Currently available evidence from direct comparisons suggests that interventions aiming to improve social cognition in schizophrenia should consider the importance of concomitant cognitive impairments and clinical symptoms, which should be adequately addressed in order to maximize gains from the interventions aimed at social cognitive skills. Lindenmayer et al. have previously shown that the combination of cognitive remediation with social cognition training is associated with better intervention outcomes than cognitive remediation alone (72). Conversely, social cognitive interventions will probably lead to better results when associated with cognitive remediation. In ASD, social cognitive interventions should probably aim at improving the recognition and integration of social stimuli to boost social motivation rather than focus on specific social skills (68).

## Limitations

Interpretation of our results should be made bearing in mind the significant heterogeneity we found in our analyzes. Such heterogeneity may be related to the use of different measures to assess the same social cognition dimensions, but also to the high variability in study populations, particularly in terms of participants' age, gender, and included diagnoses. Moreover, sample sizes were often small (*n* < 30), a frequent feature of social cognition studies. Such limitations are further compounded by the inevitable uncertainty intrinsic to attempts at meta-analyzing studies in such a broad and subtly complex field as social cognition, marred by an apparent infinity of measurement tools and concepts whose similarities and boundaries are not always clear. Notwithstanding, we opted to conduct a meta-analysis of direct comparisons between participants with schizophrenia and ASD rather than a solely descriptive review, based on the following reasons: (1) several meta-analyses have been conducted in the past regarding social cognition in patients with schizophrenia (73, 74), ASD (75, 76), and indirectly comparing both disorders (11–13); (2) our primary aim was to look at the differences in social cognitive impairments between schizophrenia and ASD, and not at social cognitive performance per se, and direct comparisons have been previously highlighted in the literature as a valuable approach to do this (1); (3) although some differences can be found in the operationalization of social cognitive domains in schizophrenia and ASD, there are common dimensions like emotion perception, ToM and social skills, that allow for the collection of data from both groups using the same or psychometrically related measures; (4) although studies are generally small, we identified a relevant number of studies evaluating the same social cognitive domains; and (5) meta-analytical methods allow for the investigation of the moderator effect of variables such as age. Indeed, moderator analysis of the effects of age on effect sizes found that for some aspects of social cognition differences between ASD and schizophrenia are critically dependent on participants' age, decreasing with increasing age. This means that studies where the schizophrenia group is significantly older than the ASD group are likely to under-estimate differences across the two groups, and future studies must strive to match the participants in each group regarding this variable. Other potentially confounding factors are participant IQ, gender, and psychiatric comorbidities, that more often than not are not equally distributed across the two diagnostic groups or have not been accounted for. Finally, in the overwhelming majority of studies no mention is made of rater blinding with respect to participants' diagnostic group, thus exposing most studies to measurement bias.

## Conclusions

Studies that compared social cognitive performance in ASD and schizophrenia show that individuals with ASD perform significantly worse than individuals with schizophrenia in emotion recognition tasks, and that this difference becomes less pronounced with increasing age. With respect to other dimensions of social cognition, available evidence is contradictory, and aggregated data do not show meaningful differences between the two diagnostic groups. It is currently not clear whether this absence of significant differences reflects shared disease mechanisms or an inability of currently used instruments to detect subtle, qualitative differences. On the other hand, study heterogeneity and the complexities of assessing social cognition caveat against overstating the reliability of aggregated data analyses in this field. Future studies addressing this question should be based on larger and more homogeneous samples, and should ideally accompany the assessment of social cognitive tasks with other measures, namely neuroimaging and neurophysiologic measures such as eye-tracking or event-related potentials. Such studies will contribute to a better understanding of the mechanisms that are specific to each disorder, and will pave the way to the development of more specific and hopefully more effective therapeutic interventions aimed at improving social skills in each of these disorders.

## Author contributions

JB-C, RJ, and JF planned and designed the study. JF and RC conducted the literature search and selection of articles for the review. JF and RC extracted data from eligible studies. JF and JB-C conducted data analysis. JF, RC, RL, and JB-C were responsible for drafting the introduction, methods and results sections of the manuscript. All authors contributed equally for the discussion section and for the final review of the manuscript.

### Conflict of interest statement

The authors declare that the research was conducted in the absence of any commercial or financial relationships that could be construed as a potential conflict of interest.

## Abbreviations

ACC: anterior cingulate cortex
ASD: autism spectrum disorders
ASSI: Attributional Style Structured Interview
Cint: Communicative Intention
DMN: default mode network
ER: emotion recognition
ER-40: Penn Emotion Recognition Task
FEP: first episode psychosis
FG: fusiform gyrus
fMRI: functional magnetic resonance imaging
fNIRS: functional near-infrared spectroscopy
IQ: intelligence quotient
MASC: Movie for the Assessment of Social Cognition
MPFC: medial prefrontal cortex
MRI: magnetic resonance imaging
MSCEIT: Mayer–Salovey–Caruso Emotional Intelligence Test
NOS: not otherwise specified
NP: non-paranoid
P: paranoid
PANSS: Positive and Negative Syndrome Scale
PFC: prefrontal cortex
PhC: Physical Causality
RMET: Reading the Mind in the Eyes Test
ROI: region of interest
SCZ: schizophrenia
SD: standard deviation
SN: salience network
SPD: schizotypal personality disorder
STS: superior temporal sulcus
ToM: theory of mind
TPJ: temporo-parietal junction
VBMA: voxel based morphometry analysis
VLPFC: ventrolateral prefrontal cortex.
